# Race/Ethnicity, Underlying Medical Conditions, Homelessness, and Hospitalization Status of Adult Patients with COVID-19 at an Urban Safety-Net Medical Center — Boston, Massachusetts, 2020

**DOI:** 10.15585/mmwr.mm6927a3

**Published:** 2020-07-10

**Authors:** Heather E. Hsu, Erin M. Ashe, Michael Silverstein, Melissa Hofman, Samantha J. Lange, Hilda Razzaghi, Rebecca G. Mishuris, Ravin Davidoff, Erin M. Parker, Ana Penman-Aguilar, Kristie E.N. Clarke, Anna Goldman, Thea L. James, Karen Jacobson, Karen E. Lasser, Ziming Xuan, Georgina Peacock, Nicole F. Dowling, Alyson B. Goodman

**Affiliations:** ^1^Boston Medical Center, Boston, Massachusetts; ^2^Division of General Academic Pediatrics, Department of Pediatrics, Boston University School of Medicine, Boston, Massachusetts; ^3^CDC COVID-19 Response Team; ^4^Section of General Internal Medicine, Department of Medicine, Boston University School of Medicine, Boston, Massachusetts; ^5^Section of Infectious Diseases, Department of Medicine, Boston University School of Medicine, Boston, Massachusetts; ^6^Department of Community Health Sciences, Boston University School of Public Health, Boston, Massachusetts.

As of July 5, 2020, approximately 2.8 million coronavirus disease 2019 (COVID-19) cases and 130,000 COVID-19–associated deaths had been reported in the United States (*1*). Populations historically affected by health disparities, including certain racial and ethnic minority populations, have been disproportionally affected by and hospitalized with COVID-19 (*2*–*4*). Data also suggest a higher prevalence of infection with SARS-CoV-2, the virus that causes COVID-19, among persons experiencing homelessness (*5*). Safety-net hospitals,^†^ such as Boston Medical Center (BMC), which provide health care to persons regardless of their insurance status or ability to pay, treat higher proportions of these populations and might experience challenges during the COVID-19 pandemic. This report describes the characteristics and clinical outcomes of adult patients with laboratory-confirmed COVID-19 treated at BMC during March 1–May 18, 2020. During this time, 2,729 patients with SARS-CoV-2 infection were treated at BMC and categorized into one of the following mutually exclusive clinical severity designations: exclusive outpatient management (1,543; 56.5%), non-intensive care unit (ICU) hospitalization (900; 33.0%), ICU hospitalization without invasive mechanical ventilation (69; 2.5%), ICU hospitalization with mechanical ventilation (119; 4.4%), and death (98; 3.6%). The cohort comprised 44.6% non-Hispanic black (black) patients and 30.1% Hispanic or Latino (Hispanic) patients. Persons experiencing homelessness accounted for 16.4% of patients. Most patients who died were aged ≥60 years (81.6%). Clinical severity differed by age, race/ethnicity, underlying medical conditions, and homelessness. A higher proportion of Hispanic patients were hospitalized (46.5%) than were black (39.5%) or non-Hispanic white (white) (34.4%) patients, a finding most pronounced among those aged <60 years. A higher proportion of non-ICU inpatients were experiencing homelessness (24.3%), compared with homeless patients who were admitted to the ICU without mechanical ventilation (15.9%), with mechanical ventilation (15.1%), or who died (15.3%). Patient characteristics associated with illness and clinical severity, such as age, race/ethnicity, homelessness, and underlying medical conditions can inform tailored strategies that might improve outcomes and mitigate strain on the health care system from COVID-19.

All adult patients who had a positive reverse transcription–polymerase chain reaction test result for SARS-CoV-2 in ambulatory or inpatient settings at BMC during March 1–May 18, 2020, were included in the analysis. SARS-CoV-2 testing was requisitioned by treating clinicians who were following guidance from the Massachusetts Department of Public Health^§^ (*6*). Data on patient age, sex, race/ethnicity, underlying medical conditions, living situation (including homelessness or residing in a nursing home), and clinical status were extracted from BMC’s electronic health records. The study was reviewed by the Boston Medical Center and Boston University Medical Campus Institutional Review Board and received a designation of nonhuman subjects research; no identifying information was extracted from the electronic health record because all data were extracted as aggregate counts. Data were collected as part of public health response activities and were determined by CDC not to constitute human subject research.^¶^Patient outcomes were assigned to one of five mutually exclusive categories designed to reflect each patient’s highest level of COVID-19 clinical severity: exclusive outpatient management, non-ICU inpatient hospitalization, ICU hospitalization without mechanical ventilation, ICU with mechanical ventilation, and all-cause death that occurred in any location (inpatient or otherwise). Hospitalization status as of May 18, 2020, and the highest level of care received by those who died were also determined. All patients who died had been hospitalized; for this analysis, exclusive outpatient management and all categories of hospitalization refer to cases that did not result in death. Underlying medical conditions were defined using *International Classification of Diseases, Tenth Revision* codes from patients’ active condition lists or encounter diagnoses within the electronic health record. Obesity was defined as body mass index ≥30 kg/m^2^. Homelessness was identified by an encounter registration screening question, use of an inpatient homeless discharge planning service, or registration address listed as a known homeless shelter. Clinical outcomes were examined by demographic characteristics, underlying medical conditions, and living situation. All analyses are descriptive, and no statistical testing was performed.

Among 2,729 patients with laboratory-confirmed COVID-19, 928 (34.0%) were aged ≥60 years, and 1,417 (51.9%) were female (Table 1). Race/ethnicity was known for 91.3% of patients, including 44.6% who identified as black, 30.1% as Hispanic, 13.5% as white, and 3.1% as another race/ethnicity. Overall, approximately one half of all patients (1,543; 56.5%) were managed exclusively as outpatients; 1,088 (39.9%) were hospitalized, including 900 (33.0%) who received non-ICU inpatient care, 69 (2.5%) who received ICU care without mechanical ventilation, 119 (4.4%) who received ICU care with mechanical ventilation, and 98 (3.6%) who died. As of May 18, 2020, among 1,088 hospitalized patients, 104 (9.6%) remained hospitalized. Among 984 patients discharged after hospitalization, 140 (14.2%) were discharged to a BMC-affiliated COVID-19 respite center which opened on April 9, 2020, for persons unable to self-isolate during the post-discharge recovery period.

**TABLE 1:** Clinical characteristics of patients with COVID-19 (N = 2,729) — Boston Medical Center, March 1–May 18, 2020

Characteristic†	Total (N = 2,729)	Mutually exclusive clinical severity categories				
		Outpatient management (n = 1,543)	Inpatient hospitalization*			Deceased**^§^** (n = 98)
			Non-ICU (n = 900)	ICU without mechanical ventilation (n = 69)	ICU with mechanical ventilation (n = 119)	
	No. (%)					
**Age group (yrs)**						
18–29	**309 (11.3)**	244 (15.8)	53 (5.9)	3 (4.3)	9 (7.6)	0 (—)
30–39	**472 (17.3)**	325 (21.1)	125 (13.9)	6 (8.7)	11 (9.2)	5 (5.1)
40–49	**503 (18.4)**	322 (20.9)	149 (16.6)	9 (13.0)	17 (14.3)	6 (6.1)
50–59	**517 (18.9)**	281 (18.2)	187 (20.8)	14 (20.3)	28 (23.5)	7 (7.1)
60–69	**460 (16.9)**	207 (13.4)	176 (19.6)	17 (24.6)	30 (25.2)	30 (30.6)
70–79	**258 (9.5)**	82 (5.3)	126 (14.0)	11 (15.9)	19 (16.0)	20 (20.4)
≥80	**210 (7.7)**	82 (5.3)	84 (9.3)	9 (13.0)	5 (4.2)	30 (30.6)
**Sex**						
Female	**1,417 (51.9)**	896 (58.1)	428 (47.6)	21 (30.4)	40 (33.6)	32 (32.7)
Male	**1,312 (48.1)**	647 (41.9)	472 (52.4)	48 (69.6)	79 (66.4)	66 (67.3)
**Race/Ethnicity**						
Black, non-Hispanic	**1,218 (44.6)**	689 (44.7)	399 (44.3)	32 (46.4)	50 (42.0)	48 (49.0)
Hispanic or Latino	**821 (30.1)**	421 (27.3)	320 (35.6)	19 (27.5)	43 (36.1)	18 (18.4)
White, non-Hispanic	**369 (13.5)**	221 (14.3)	101 (11.2)	10 (14.5)	16 (13.4)	21 (21.4)
Other race, non-Hispanic^¶^	**84 (3.1)**	60 (3.9)	17 (1.9)	2 (2.9)	2 (1.7)	3 (3.1)
Unknown/Declined	**237 (8.7)**	152 (9.9)	63 (7.0)	6 (8.7)	8 (6.7)	8 (8.2)
Underlying medical conditions**						
Asthma	**360 (13.2)**	176 (11.4)	140 (15.6)	6 (8.7)	23 (19.3)	15 (15.3)
Cancer	**195 (7.1)**	67 (4.3)	90 (10.0)	10 (14.5)	10 (8.4)	18 (18.4)
Chronic kidney disease	**332 (12.2)**	115 (7.5)	149 (16.6)	13 (18.8)	20 (16.8)	35 (35.7)
Chronic kidney disease on dialysis	**106 (3.9)**	31 (2.0)	53 (5.9)	5 (7.2)	8 (6.7)	9 (9.2)
Cirrhosis	**42 (1.5)**	17 (1.1)	16 (1.8)	2 (2.9)	3 (2.5)	4 (4.1)
Congestive heart failure	**216 (7.9)**	59 (3.8)	106 (11.8)	8 (11.6)	11 (9.2)	32 (32.7)
Chronic obstructive pulmonary disease	**146 (5.3)**	35 (2.3)	78 (8.7)	6 (8.7)	11 (9.2)	16 (16.3)
Coronary artery disease	**190 (7.0)**	71 (4.6)	73 (8.1)	6 (8.7)	10 (8.4)	30 (30.6)
Diabetes	**708 (25.9)**	274 (17.8)	317 (35.2)	24 (34.8)	47 (39.5)	46 (46.9)
HIV/AIDS	**73 (2.7)**	36 (2.3)	31 (3.4)	2 (2.9)	2 (1.7)	2 (2.0)
Hypertension	**1,248 (45.7)**	556 (36.0)	516 (57.3)	39 (56.5)	66 (55.5)	71 (72.4)
Obesity (BMI >30 kg/m^2^)	**1,164 (42.7)**	553 (35.8)	465 (51.7)	31 (44.9)	69 (58.0)	46 (46.9)
Serious mental illness	**219 (8.0)**	87 (5.6)	103 (11.4)	7 (10.1)	13 (10.9)	9 (9.2)
Sickle cell disease	**15 (0.5)**	5 (0.3)	8 (0.9)	0 (—)	1 (0.8)	1 (1.0)
Substance use disorder	**396 (14.5)**	161 (10.4)	178 (19.8)	14 (20.3)	24 (20.2)	19 (19.4)
≥1 of above conditions	**2,033 (74.5)**	977 (63.3)	799 (88.8)	57 (82.6)	111 (93.3)	89 (90.8)
≥2 of above conditions	**1,429 (52.4)**	606 (39.3)	613 (68.1)	44 (63.8)	89 (74.8)	77 (78.6)
**Living situation^††^**						
Homelessness	**447 (16.4)**	184 (11.9)	219 (24.3)	11 (15.9)	18 (15.1)	15 (15.3)
Residing in nursing home	**181 (6.6)**	114 (7.4)	44 (4.9)	6 (8.7)	7 (5.9)	10 (10.2)
**Pregnant** ^§§^	**89 (3.3)**	42 (2.7)	42 (4.7)	1 (1.4)	4 (3.4)	0 (—)

**Abbreviations:** AIDS = acquired immunodeficiency syndrome; BMI = body mass index; COVID-19 = coronavirus disease 2019; HIV = human immunodeficiency virus; ICU = intensive care unit.

* Survived.

^†^ Patient characteristics are not mutually exclusive; therefore, the counts and proportions might not sum to the totals.

^§^ Of the 98 patients who died, all had been hospitalized, including 27 (27.6%) who received non-ICU inpatient care, 15 (15.3%) who received ICU care without mechanical ventilation, and 56 (57.1%) who received ICU care with mechanical ventilation

^¶^ Other race included persons who identified as Asian, American Indian, Middle Eastern, Native Hawaiian/Pacific Islander. These groups were consolidated due to small numbers.

** Underlying medical conditions were defined using *International Classification of Diseases, Tenth Revision* codes from patients’ active condition lists or encounter diagnoses within the electronic health record. Obesity was defined by BMI ≥30 kg/m^2^. Patients with substance use disorder were additionally identified via presence of orders for inpatient assessment of opiate or alcohol withdrawal symptoms, inpatient consult to an addiction medicine service, or encounters for previous outpatient substance use disorder treatment.

^††^ Homelessness was identified by a registration screening question, use of an inpatient homeless discharge planning service, or registration address listed as a known homeless shelter. Nursing home residence was identified by cross-referencing a list of known nursing home patients or matching registration address with known nursing home addresses.

^§§^ Patients were categorized as pregnant if a health care encounter for COVID-19 occurred before, or up to 7 days after, the end of pregnancy.

Older age, male sex, and having one or more underlying medical conditions were more prevalent among patients who were hospitalized or died (Table 1). For example, patients aged ≥60 years accounted for 24.0% (371 of 1,543) of outpatients, but 81.6% (80 of 98) of deaths. In addition, whereas 63.3% of outpatients had one or more underlying medical conditions, 93.3% of those who received mechanical ventilation and 90.8% of those who died had one or more underlying conditions. A higher proportion of black patients had one or more (80.7%) or two or more (61.2%) underlying conditions than did other racial and ethnic groups, whereas a higher proportion of white patients were aged ≥80 years (13.0%) (Table 2). The prevalence of homelessness was higher among those who experienced non-ICU hospitalization (24.3%) than among those who experienced more severe clinical outcomes: prevalence of homelessness was 15.9% among ICU hospitalizations without mechanical ventilation, 15.1% among ICU hospitalizations with mechanical ventilation, and 15.3% among those who died (Table 1).

**TABLE 2:** Characteristics of patients with COVID-19 by race/ethnicity (N = 2,729) — Boston Medical Center, March 1–May 18, 2020

Characteristics*	Race/Ethnicity					
	Total (N = 2,729)	Black, non-Hispanic (n = 1,218)	Hispanic/Latino (n = 821)	White, non-Hispanic (n = 369)	Other race, non-Hispanic^†^ (n = 84)	Unknown/Declined (n= 237)
	No. (%)					
**Age group (yrs)**						
18–29	**309 (11.3)**	106 (8.7)	129 (15.7)	26 (7.0)	13 (15.5)	35 (14.8)
30–39	**472 (17.3)**	198 (16.3)	152 (18.5)	67 (18.2)	13 (15.5)	42 (17.7)
40–49	**503 (18.4)**	213 (17.5)	190 (23.1)	46 (12.5)	15 (17.9)	39 (16.5)
50–59	**517 (18.9)**	223 (18.3)	165 (20.1)	66 (17.9)	15 (17.9)	48 (20.3)
60–69	**460 (16.9)**	232 (19.0)	112 (13.6)	69 (18.7)	10 (11.9)	37 (15.6)
70–79	**258 (9.5)**	137 (11.2)	46 (5.6)	47 (12.7)	8 (9.5)	20 (8.4)
≥80	**210 (7.7)**	109 (8.9)	27 (3.3)	48 (13.0)	10 (11.9)	16 (6.8)
**Sex**						
Female	**1,417 (51.9)**	657 (53.9)	389 (47.4)	185 (50.1)	49 (57.1)	137 (57.8)
Male	**1,312 (48.1)**	561 (46.1)	432 (52.6)	184 (49.9)	35 (41.7)	100 (42.2)
**Underlying medical conditions** ^§^						
Asthma	**360 (13.2)**	188 (15.4)	102 (12.4)	43 (11.7)	6 (7.1)	21 (8.9)
Cancer	**195 (7.1)**	106 (8.7)	43 (5.2)	31 (8.4)	4 (4.8)	11 (4.6)
Chronic kidney disease	**332 (12.2)**	222 (18.2)	55 (6.7)	34 (9.2)	7 (8.3)	14 (5.9)
Chronic kidney disease on dialysis	**106 (3.9)**	64 (5.3)	22 (2.7)	10 (2.7)	3 (3.6)	7 (3.0)
Cirrhosis	**42 (1.5)**	20 (1.6)	10 (1.2)	8 (2.2)	0 (0.0)	4 (1.7)
Congestive heart failure	**216 (7.9)**	129 (10.6)	32 (3.9)	44 (11.9)	3 (3.6)	8 (3.4)
Chronic obstructive pulmonary disease	**146 (5.3)**	70 (5.7)	16 (1.9)	47 (12.7)	4 (4.8)	9 (3.8)
Coronary artery disease	**190 (7.0)**	104 (8.5)	35 (4.3)	40 (10.8)	2 (2.4)	9 (3.8)
Diabetes mellitus	**708 (25.9)**	382 (31.4)	196 (23.9)	53 (14.4)	21 (25.0)	56 (23.6)
HIV/AIDS	**73 (2.7)**	47 (3.9)	11 (1.3)	8 (2.2)	0 (0.0)	7 (3.0)
Hypertension	**1,248 (45.7)**	686 (56.3)	292 (35.6)	149 (40.4)	28 (33.3)	93 (39.2)
Obesity (BMI ≥30 kg/m^2^)	**1,164 (42.7)**	576 (47.3)	388 (47.3)	102 (27.6)	11 (13.1)	87 (36.7)
Serious mental illness	**219 (8.0)**	89 (7.3)	57 (6.9)	59 (16.0)	8 (9.5)	6 (2.5)
Sickle cell disease	**15 (0.5)**	11 (0.9)	3 (0.4)	0 (0.0)	0 (0.0)	1 (0.4)
Substance use disorder	**396 (14.5)**	171 (14.0)	98 (11.9)	105 (28.5)	8 (9.5)	14 (5.9)
≥1 of above conditions	**2,033 (74.5)**	983 (80.7)	602 (73.3)	258 (69.9)	43 (51.2)	147 (62.0)
≥2 of above conditions	**1,429 (52.4)**	745 (61.2)	366 (44.6)	193 (52.3)	30 (35.7)	95 (40.1)
**Living situation^¶^**						
Homelessness	**447 (16.4)**	203 (16.7)	100 (12.2)	110 (29.8)	11 (13.1)	23 (9.7)
Residing in nursing home	**181 (6.6)**	101 (8.3)	14 (1.7)	51 (13.8)	11 (13.1)	4 (1.7)
Pregnant**	**89 (3.3)**	30 (2.5)	49 (6.0)	4 (1.1)	2 (2.4)	4 (1.7)

**Abbreviations:** AIDS = acquired immunodeficiency syndrome; BMI = body mass index; COVID-19 = coronavirus disease 2019; HIV = human immunodeficiency virus.

* Patient characteristics are not mutually exclusive; therefore, the counts and proportions might not sum to totals.

^†^ Other race included persons who identified as Asian, American Indian, Middle Eastern, Native Hawaiian/Pacific Islander. These groups were consolidated because of small numbers.

^§^ Underlying medical conditions were defined using *International Classification of Diseases, Tenth Revision* codes from patients’ active condition lists or encounter diagnoses within the electronic health record. Obesity was defined by BMI ≥30 kg/m^2^. Patients with substance use disorder were additionally identified via presence of orders for inpatient assessment of opiate or alcohol withdrawal symptoms, inpatient consult to an addiction medicine service, or encounters for previous outpatient substance use disorder treatment.

^¶^ Homelessness was identified by a registration screening question, use of an inpatient homeless discharge planning service, or registration address listed as a known homeless shelter. Nursing home residence was identified by cross-referencing a list of known nursing home patients or matching registration address with known nursing home addresses.

** Patients were categorized as pregnant if a health care encounter for COVID-19 occurred before, or up to 7 days after, the end of pregnancy.

The clinical severity of illness among patients with COVID-19 varied by race/ethnicity and age. Overall, the hospitalization rate was higher among Hispanic patients (382 of 821, 46.5%) than among black (481 of 1,218; 39.5%) or white (127 of 369; 34.4%) patients (Figure). In particular, among patients aged <60 years, 43.2% (275 of 636) of Hispanic patients were hospitalized, compared with 30.8% (228 of 740) of black patients and 29.8% (61 of 205) of white patients. Although the highest number of deaths occurred among black patients, the highest percentage of deaths occurred among white patients (21 of 369; 5.7%), compared with black (48 of 1,218; 3.9%) and Hispanic (18 of 821; 2.2%) patients. Among patients aged ≥60 years, 11.0% of white, 9.0% of black, and 5.4% of Hispanic patients died.

**FIGURE:**
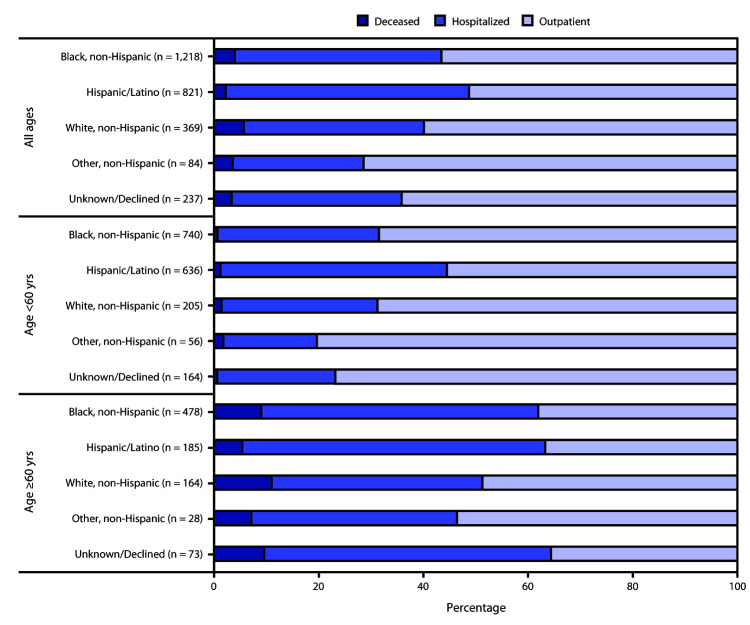
Clinical severity of illness in patients* with COVID-19, by age and race/ethnicity (N = 2,729) — Boston Medical Center, March 1–May 18, 2020 **Abbreviation:** COVID-19 = coronavirus disease 2019. * Inpatients include surviving patients whose highest level of care included non–intensive care unit hospitalization or intensive care unit hospitalization with or without invasive mechanical ventilation.

## Discussion

Among 2,729 COVID-19 patients cared for in inpatient and outpatient settings at BMC during March 1–May 18, nearly one half were black, approximately one third were Hispanic, and one in six were experiencing homelessness. Compared with black or white patients, a higher proportion of Hispanic patients were hospitalized; this finding was most notable among persons aged <60 years. Approximately one in five patients hospitalized at BMC were experiencing homelessness. The overall case-fatality rate was higher among white patients than among black or Hispanic patients; this finding is potentially explained by higher proportions of white patients in the oldest age groups, which are at highest risk for COVID-19–associated complications and death (*2*,*4*).

Long-standing systemic health, health care, and socioeconomic inequities and systemic racism, which influence life expectancy, underlying medical conditions, and health care access and utilization, as well as current work and living circumstances are all factors that can play a crucial role in risk for COVID-19 exposure, illness, and mortality (*7*,*8*). Although this report was unable to fully assess the associations between these factors and COVID-19 outcomes, the findings reflect the experience of a safety-net institution within a city that experienced a surge in COVID-19 cases during April 2020 and whose patients historically include high proportions of persons at increased risk for adverse health outcomes (including racial and ethnic minority groups and persons experiencing homelessness). At BMC, information about individual patients’ living situations, family structures, and economic means factored into care teams’ hospitalization and discharge decisions. For example, clinicians’ concerns about patients’ inability to self-isolate resulted in decisions to lengthen inpatient hospitalizations (personal communication, Christopher Manessah, MD, and Deanna Faretra, BMC, April 2020). BMC also implemented multiple strategies to help patients who were not severely ill avoid prolonged hospitalization, including transformation of a nearby vacant hospital building into a COVID-19 recovery center for patients whose living circumstances, including homelessness, precluded their ability to self-isolate. Additional programs included home delivery of groceries or prepared meals from the BMC food pantry, provision of mobile telephones to facilitate follow-up telehealth visits, and bedside and home delivery of outpatient medications. An assessment of the effectiveness of specific strategies to support COVID-19 patients in recovery, particularly for those with health-related social needs that present barriers to hospital discharge or self-isolation, is needed.

The findings in this report are subject to at least five limitations. First, the report describes a single institution’s experience and might not be generalizable to other institutions or locations. Second, because all data were extracted as aggregate counts, statistical comparisons were not performed, and associations cannot be interpreted as being statistically significant, nor can causality be inferred. Third, approximately 4% of patients included in this report remained hospitalized at the end of data collection; it is unknown whether these patients have meaningfully different characteristics relative to the larger study population. Comprehensive external vital statistics were unavailable; out-of-hospital deaths, although assessed, were potentially undercounted. Fourth, intermittent shortages of testing supplies introduced changes to BMC’s testing criteria throughout the study period, which might have influenced whether patients were tested, particularly in outpatient settings. Finally, this report uses location of care, mechanical ventilation status, and death to categorize patients into clinical severity categories, which might discount the role of contextual factors that influence care received, including availability of critical care beds, evolving clinical practice, and patient preferences (e.g., advance directives).

Experience treating COVID-19 patients at a single safety-net institution highlighted associations between clinical outcomes and sociodemographic characteristics, including age, race/ethnicity, underlying medical conditions, and homelessness. One important strength of this report is that data on race and ethnicity, which are often incomplete in public reports (*9*), were available for 91.3% of the patients and are presented by age category. Further study is needed to assess the impact of BMC’s strategies for addressing health-related social needs of patients with COVID-19 on related health outcomes and health care utilization, and to understand how these characteristics can inform development of tailored strategies that might improve patient outcomes and alleviate strain on the health care system.

**Summary**

**What is already known about this topic?**

Older adults and non-Hispanic black and Hispanic persons are overrepresented among hospitalized COVID-19 patients in the United States. High COVID-19 prevalence has been reported among residents of homeless shelters.

**What is added by this report?**

During March–May 2020, among 2,729 COVID-19 patients treated at an urban safety-net hospital serving predominantly low-income racial/ethnic minority populations, clinical severity differed by age, race/ethnicity, underlying medical conditions, and homelessness. Hospitalized patients were more likely to be Hispanic or to be experiencing homelessness; >80% of patients who died were aged ≥60 years.

**What are the implications for public health practice?**

COVID-19 patient characteristics, including age, race/ethnicity, and homelessness could inform tailored strategies that might improve patient outcomes and mitigate strain on health care systems.

## Footnotes

* These authors contributed equally.

† https://www.ncbi.nlm.nih.gov/books/NBK401306.

§ Guidance from Massachusetts Department of Public Health (MADPH) on clinical and epidemiologic criteria for molecular SARS-CoV-2 testing evolved throughout the study period. Treating clinicians required approval from MADPH to requisition tests until March 15, 2020. In-hospital testing became available at Boston Medical Center on March 24, 2020, and routine testing of all hospitalized patients began on April 27, 2020. MADPH recommended routine molecular testing of persons identified as close contacts of patients with confirmed COVID-19 beginning on May 11, 2020.

¶ https://www.ecfr.gov/cgi-bin/retrieveECFR?gp=&SID=83cd09e1c0f5c6937cd9d7513160fc3f&pitd=20180719&n=pt45.1.46&r=PART&ty=HTML.
